# Comparison of MALDI-TOF MS instruments and databases for the identification of uncommon yeasts, *Aspergillus* spp. and rare filamentous fungi

**DOI:** 10.1128/jcm.01612-24

**Published:** 2025-05-15

**Authors:** Marion Dutkiewicz, Maéva Garros, Julie Bui, Véronique Charlier, Elodie Da Silva, Maryline Lemaire, Sarah Dellière, Anne-Cécile Normand, Renaud Piarroux, Samia Hamane, Théo Ghelfenstein-Ferreira, Alexandre Alanio

**Affiliations:** 1 Laboratoire de Parasitologie-Mycologie, AP-HP, Hôpital Saint-Louis 55663, Paris, Île-de-France, France; 2 Centre National de Référence Mycoses Invasives et Antifongiques, Institut Pasteur, Université Paris Cité 555089, Paris, Île-de-France, France; 3 Groupe de recherche Mycologie Translationnelle, Institut Pasteur, Université Paris Cité 555089, Paris, Île-de-France, France; 4 Département de Mycologie, Institut Pasteur, Université Paris Cité 555089, Paris, Île-de-France, France; 5 Laboratoire de Parasitologie-Mycologie, Hôpital de la Pitié-Salpétrière, APHP 727386 , Tunis, Tunis, Tunisia; University of Calgary, Calgary, Alberta, Canada

**Keywords:** Maldi-TOF, identification, yeasts, molds, filamentous fungi, *Aspergillus*, section, MSI2

## Abstract

Identification of uncommon fungi using matrix-assisted laser desorption/ionization time-of-flight mass spectrometry (MALDI-ToF-MS) remains challenging. Its performance depends on the protein extraction method, instrument, algorithm, and database. This study compared two established mass spectrometers (VITEK MS [bioMérieux] and Microflex [Bruker]) to the new VITEK MS PRIME (bioMérieux) and four databases, including MSI-2, a non-commercial database constructed with spectra acquired with Bruker instruments. Isolates of *Aspergillus* species, rare molds, and uncommon yeasts, previously identified by sequencing, were analyzed. After a two-step protein extraction, MALDI-ToF-MS identification was performed, and spectra were submitted to *in vitro* diagnostic (IVD) (KB3.2 and KB3.3 [bioMérieux]), research use only (RUO) (FilFungi V5 [Bruker]) databases and MSI-2. A total of 169 isolates (61 *Aspergillus* spp., 72 rare molds, and 36 uncommon yeasts), representing 33 genera and 96 species, were included. Identification rates at species level for all fungi were similar between VITEK MS and VITEKMS PRIME (79% vs. 77%). The main difference lies in the rates of non-analyzable spectra, being 15% (26 strains) for VITEK MS PRIME versus only 3% (5 strains) for VITEK MS. For *Aspergillus* and rare molds, MSI-2 performed equally well on VITEK MS and Microflex (92% vs. 91%), indicating a spectra compatibility. For uncommon yeasts, all databases performed equally at the species level. For *Aspergillus* and rare molds, FilFungi V5 performed poorly (23% and 21%), while MSI-2 was best (77% and 82%) due to broader species coverage. Misidentifications mostly involved cryptic *Aspergillus* species. MALDI-ToF-MS is a powerful tool for identifying rare fungi. Improvements are needed in completing commercial databases and optimizing acquisition systems for fungal spectra.

**IMPORTANCE:**

Matrix-assisted laser desorption/ionization time-of-flight mass spectrometry (MALDI-ToF-MS) is key for fungal identification nowadays. We present here the largest comparison of MALDI-ToF-MS instruments and databases focusing on rare yeasts and molds identification, challenging the existing instruments and both commercial and academic databases. The strains were collected in Saint Louis Hospital (Paris) and identified using bar-code sequencing. We showed that commercial databases are efficient for the identification of the main fungal species tested, whereas the academic database outperforms them for the identification of cryptic species.

## INTRODUCTION

Matrix-assisted laser desorption/ionization time-of-flight (MALDI-ToF) is nowadays widely used in microbial diagnostics to identify bacteria (1), and it has recently been proven as a more accurate way to identify yeasts than the former biochemical methods (2). However, MALDI-ToF identification of filamentous fungi (FF) remains a challenge due to multiple factors influencing the results, such as the age of the colony, type of matrix used, incubation temperature, and culture medium (3).

Unlike bacteria, FF are made of a thick cell wall made of polymers of sugars such as glucans and chitin that must be lysed to liberate the cytoplasmic proteins used to create the mass spectrometer (MS) spectrum; thus, the protein extraction method is a key step. In 2011, Cassagne et al*.* (4) validated the method that is now the most widely used by both manufacturers and university laboratories.

Fungal spectra contain fewer and less intense peaks than those generated from bacteria. As such, the acquisition method has to be optimized to produce analyzable and reliable spectra with higher laser intensity and more laser impacts on the target spots (5).

The spectrum is eventually compared to a database, the comparing algorithm and the database being both limiting factors. The algorithm depends on the construction strategy of the database, which widely differs from one manufacturer to another, as already described (6).

To date, several databases are available. BioMérieux (Marcy l’Etoile, France) currently markets their european conformity (CE) *in vitro* diagnostic (IVD) Knowledge Base (KB) 3.2 and its upgrade CE IVD KB3.3; they also provide a research use only (RUO) database, SARAMIS 4.17 (Anagnostec), used only in an experimental setting. Bruker (Billerica, USA) markets their CE IVD V12 database which contains yeast species plus a RUO database, FilFungi V5, which integrates mold reference spectra. A non-commercial database, MSI-2, has also been made available online for free where the user submits their spectrum in the software and retrieves its results within minutes (7).

MALDI-ToF identification for molds could be of valuable help for medical mycologists. In many laboratories, mold identification still relies on the microscopic visualization of conidia and conidiogenous cells, and such a method requires a high degree of expertise. Results are available within minutes but can be limited to the genus or section level with several cryptic species known to be phenotypically indistinguishable. In those cases, sequencing can complete the identification, but the results can be delayed, and the cost is significant. On the other hand, MALDI-ToF MS allows a fast and cheap investigation at the species level of multiple colonies harbouring different phenotypical characteristics from the culture plate.

This study aims at comparing the performances of two commercial MS (VITEK MS [bioMérieux, Marcy l’Etoile, France] and Microflex [Bruker, Billerica, USA]) and the recent VITEK MS PRIME (bioMérieux), which allows a faster technical workflow and is associated with a new acquisition system for the identification of uncommon yeasts, *Aspergillus* spp., and rare molds. In addition to the instruments, four databases (KB3.2, KB3.3, FilFungi V5 [Bruker], and MSI-2 [8]) were also compared for fungal identification.

## MATERIALS AND METHODS

### Strain collection

The strains were collected from our hospital storage at −80°C, corresponding to pathogenic species, isolated from clinical and environmental samples between 2007 and 2012. Aiming at the widest diversity, three isolates per occurring species, when possible, were included. A total of 169 isolates were analyzed, including 61 *Aspergillus* spp., 72 rare molds, and 36 uncommon yeasts, representing 33 genera and 96 species (Table S1).

### Strain culture

The strains were thawed from the −80°C freezer and sub-cultured on Sabouraud Chloramphenicol Gentamicin agar (Bio-Rad, Marnes-La-Coquette, France) at 30°C. Identification was performed at day 3 (D3) and D8 of growth using the same plate for both time points.

### Sequencing

All fungal identifications were validated by Sanger sequencing using the relevant barcoding genes for a given genus. *Aspergillus* spp. strains were identified using beta-tubulin and calmodulin. *Paecilomyces*/*Penicillium*/*Talaromyces*/*Scedosporium* strains were identified using beta-tubulin and V9D-LS266 (extended Internal Transcribed Spacer [ITS] region) (9). All other genera were identified using V9D-LS266.

Isolates were cultured on Sabouraud Chloramphenicol Gentamicin Agar (Bio-Rad, Marnes-La-Coquette, France). DNA extraction was performed using the EZ1 (Qiagen, Hilden, Germany) and extraction quality verified with a Qubit 3 Fluorometer (Invitrogen, Waltham, USA). Sanger sequencing was conducted on the 3500xL (Applied Biosystems, Waltham, USA). The raw sequences were trimmed manually on the Geneious software (Dotmatics, Boston, USA). The definitive identification was established upon submission to the Mycobank curated database (www.mycobank.org).

### Extraction procedure

For *Aspergillus* spp. and rare molds, a two-step protein extraction (4) was performed using the VITEK MS MOULD KIT. The fungal colony (1–2 cm^2^) was sampled with a moistened cotton bud and introduced in a 2-mL Eppendorf containing 900 µL of 70% ethanol and vortexed. After 2 minutes of centrifugation at 14,000 g, ethanol was fully removed from the fungal pellet, and a total of 40 µL of 70% formic acid was added and the pellet vortexed, before adding 40 µL of absolute acetonitrile. After 2 minutes of centrifugation at 14,000 g, 1 µL of supernatant was dispensed on MS target (VITEK MS-DS SLIDE or MBT Biotarget 96) and covered with 1 µL of MS matrix (MS Matrix CHCA or HCCA Matrix).

For uncommon yeasts, as bioMérieux recommends to perform a direct transfer technique, the colonies were directly spotted using a VITEK PICKME pen (BioMérieux, Marcy l’Etoile, France) on the MS target and covered with 1 µL of 33% formic acid (VITEK MS-FA, BioMérieux, Marcy l’Etoile, France) and 1 µL of MS matrix before spectra acquisition with VITEK MS and VITEK MS PRIME. Without recommendations from Bruker, a complete extraction procedure was performed, as described above, before spectra acquisition with Microflex. Of note, the MSI-2 database was not built using spectra from direct deposition of yeast colonies but rather after complete extraction (7).

### MALDI-ToF identification

For *Aspergillus* spp. and rare molds, the same extract was used for identification in duplicate on the 3MS (VITEK MS, VITEK MS PRIME [bioMérieux, Marcy l’Etoile, France] and Microflex [Bruker, Billerica, USA]). For Microflex, the AutoX acquisition system parameters were modified to record all the mass spectra acquired for further analysis. For bioMérieux instruments, those parameters could not be modified.

The obtained spectra were submitted to their corresponding commercial databases: IVD (KB3.2 and KB3.3 [bioMérieux]) and RUO (FilFungi V5 [Bruker]) as well as the non-commercial database MSI-2 (8). The database representativeness according to our fungal panel is presented in Table 1. All analyses were performed using the three following levels: genus, section (for *Aspergilli*), and species. For each database, the recommended thresholds were used to retain the obtained identification. At the genus level, for bioMérieux’s databases, the confidence score needs to be ≥60% associated to a discrimination level “Single choice”; for the Bruker database, a confidence score ≥ 1.7 ; and for the MSI-2 database, a confidence score ≥ 20 (8). At the species level, for bioMérieux’s databases, the confidence score needs to be ≥60% associated to a discrimination level “Single choice”; for the Bruker database, a confidence score ≥ 2.0; and for the MSI-2 database, a confidence score ≥ 20. Below those scores, we considered an absence of identification at the genus and/or species level. Some species are identified by KB3.2 and KB3.3 as a complex of two species (e.g., *Aspergillus calidoustus/ustus* or *Aspergillus ochraceus/westerdijkiae*). If the expected species was comprised in the complex, the identification was considered as correct.

**TABLE 1:** Coverage of the fungal panel in the four evaluated databases

	Total number of fungal species	Genus coverage	Species coverage
KB3.2	221	86%	54%
KB3.3	297	89%	58%
FilFungi V5	442	96%	60%
MSI-2	1,612	99%	92%

BioMérieux’s acquisition system has a quality control algorithm. Therefore, the acquired spectrum can be considered as not qualitative enough to be analyzed, and thus, the output data from the instrument is “Bad spectrum.” This output does not exist in the Microflex workflow. If the output data from a bioMérieux instrument was “Bad spectrum,” a re-extraction was performed from a new 3-day sub-culture. If an identification was obtained, we retained the identification, but if the second result was “Bad spectrum” again, we retained this output as final result.

Eventually, the duplicate yielding the highest score was retained for each database, regardless of the proposed identifications.

### Controls

The quality control strains *Aspergillus brasiliensis* NCPF 2275 and *Candida glabrata* ATCC 2950 were included in the workflow once per week.

### Statistics

Exact Fisher’s test was used to compare the performances of the different databases. The isolates for which only “Bad spectrum” output data could be obtained were excluded, as it reflects an instrumentation problem and would create a bias in the comparison of databases, explaining why the denominator for uncommon yeasts, *Aspergillus* spp., and rare molds varies from one instrument to another.

## RESULTS

### Optimum time of growth

All instruments and databases combined, the rates of correct identification (RCIs) (i.e., for which the result reached the defined identification threshold) were higher when performed at D3 (71%, 68%, and 77% at the genus, section, and species levels, respectively) compared to D8 (58%, 51%, and 53%, respectively). Consequently, for further analyses, only D3 values were retained.

### Ability to obtain an identification result

Regardless of whether the identification was correct or incorrect, it was necessary to evaluate whether each couple instrument/database was able to provide an identification for the tested isolates (Table 2).

**TABLE 2:** Among the identified strains, correctly identified strains for all instruments and databases at genus, section (for *Aspergillus* spp. only) and species levels^*b*^^,*f*^

	Database	Instrument	Identified genus *n*/*n*^*a*^ (%)	Genus *n*/*n*^*a*^ (%)	Identified species *n*/*n*^*a*^ (%)	Section *n*/*n*^*a*^ (%)	Species *n*/*n*^*a*^ (%)
Uncommon yeasts	KB3.2^*c*^	VITEK MS	30/33 (91)	29/30 (97)	30/33 (91)	NA	27/30 (90)
		VITEK MS PRIME	23/25 (92)	22/23 (96)	23/25 (92)	NA	20/23 (87)
	KB3.3^*c*^	VITEK MS	29/33 (88)	28/29 (97)	29/33 (88)	NA	26/29 (90)
		VITEK MS PRIME	23/25 (92)	22/23 (96)	23/25 (92)	NA	20/23 (87)
	FilFungi V5^*d*^	Microflex	30/36 (83)	30/30 (100)	22/36 (61)	NA	22/22 (100)
	MSI2^*e*^	VITEK MS	23/33 (70)	22/23 (96)	22/33 (67)	NA	21/22 (95)
		VITEK MS PRIME	16/25 (64)	16/16 (100)	15/25 (60)	NA	15/15 (100)
		Microflex	32/36 (89)	32/32 (100)	31/36 (86)	NA	30/31 (97)
*Aspergillus* spp.	KB3.2^*c*^	VITEK MS	47/61 (77)	47/47 (100)	47/61 (77)	47/47 (100)	27/47 (57)
		VITEK MS PRIME	42/55 (76)	42/42 (100)	42/55 (76)	42/42 (100)	26/42 (62)
	KB3.3^*c*^	VITEK MS	51/61 (84)	51/51 (100)	51/61 (84)	51/51 (100)	29/51 (57)
		VITEK MS PRIME	45/55 (82)	45/45 (100)	45/55 (82)	45/45 (100)	27/45 (60)
	FilFungi V5^*d*^	Microflex	31/61 (51)	30/31 (97)	14/61 (23)	13/14 (93)	7/14 (50)
	MSI2^*e*^	VITEK MS	60/61 (98)	60/60 (100)	60/61 (98)	60/60 (100)	46/60 (77)
		VITEK MS PRIME	50/55 (91)	50/50 (100)	50/55 (91)	50/50 (100)	36/50 (72)
		Microflex	58/61 (95)	57/58 (98)	58/61 (95)	55/58 (95)	39/58 (67)
Molds	KB3.2^*c*^	VITEK MS	35/70 (50)	35/35 (100)	35/70 (50)	NA	25/35 (71)
		VITEK MS PRIME	26/63 (41)	26/26 (100)	26/63 (41)	NA	20/26 (77)
	KB3.3^*c*^	VITEK MS	44/70 (63)	44/44 (100)	44/70 (63)	NA	33/44 (75)
		VITEK MS PRIME	35/63 (56)	35/35 (100)	35/63 (56)	NA	26/35 (74)
	FilFungi V5^*d*^	Microflex	34/72 (47)	33/34 (97)	15/72 (21)	NA	9/15 (60)
	MSI2^*e*^	VITEK MS	63/70 (90)	63/63 (100)	61/70 (87)	NA	50/61 (82)
		VITEK MS PRIME	56/63 (89)	56/56 (100)	55/63 (87)	NA	45/55 (82)
		Microflex	62/72 (86)	61/62 (98)	61/72 (85)	NA	49/61 (80)
All fungi	KB3.2^*c*^	VITEK MS	112/164 (68)	111/112 (99)	112/164 (68)	47/47 (100)	79/112 (71)
		VITEK MS PRIME	91/143 (64)	90/91 (99)	91/143 (64)	42/42 (100)	66/91 (73)
	KB3.3^*c*^	VITEK MS	124/164 (76)	123/124 (99)	124/164 (76)	51/51 (100)	88/124 (71)
		VITEK MS PRIME	103/143 (72)	102/103 (99)	103/143 (72)	45/45 (100)	73/103 (71)
	FilFungi V5^*e*^	Microflex	95/169 (56)	93/95 (98)	51/169 (30)	13/31 (93)	38/51 (75)
	MSI2^*e*^	VITEK MS	146/164 (89)	145/146 (99)	143/164 (87)	60/60 (100)	117/143 (82)
		VITEK MS PRIME	122/143 (85)	122/122 (100)	120/143 (84)	50/50 (100)	96/120 (80)
		Microflex	152/169 (90)	150/152 (99)	150/169 (89)	55/58 (95)	118/150 (79)

^*a*^ The strains for which only “Bad spectrum” results were obtained were excluded from the statistical analysis, that is, three uncommon yeasts and two RM for VITEK MS and 11 uncommon yeasts, six *Aspergillus* spp., and nine RM for VITEK MS PRIME. These output data are specific to bioMérieux instruments; consequently all 169 strains were retained for analysis of Microflex results.

^*b*^ Strains were considered as correctly identified when the confidence score reached the recommended threshold and the proposed identification matched the expected identification (obtained by sequencing the relevant barcoding gene).

^*c*^ Strains were considered unidentified when the confidence score did not reach the recommended threshold: at genus, species, and section levels: confidence level “Single choice” and confidence value > 60%.

^*d*^ Strains were considered unidentified when the confidence score did not reach the recommended threshold: at genus level confidence score > 1.80 and at species and section levels confidence score > 2.0.

^*e*^ Strains were considered unidentified when the confidence score did not reach the recommended threshold: at genus, species, and section levels: confidence score > 20.

^*f*^ NA, not applicable.

For uncommon yeasts, the highest rate of identified strains at the genus level was obtained with VITEK MS PRIME/KB3.2 and VITEK MS PRIME/KB3.3 (92%) and at the species level with VITEK MS PRIME/KB3.2 and VITEK MS PRIME/KB3.3 (92%). The lowest rates both at the genus and the species levels were obtained with VITEK MS PRIME/MSI-2 (64% and 60%, respectively). For *Aspergillus* spp., the highest rates both at the genus and the species levels were obtained with VITEK MS/MSI-2 (98% and 98%, respectively). The lowest rates were obtained with Microflex/FilFungi V5 (51% and 23%, respectively). For rare molds, the highest rates both at the genus and the species levels were obtained with VITEK MS/MSI-2 (90% and 87%, respectively). The lowest rates were obtained at the genus level with VITEK MS PRIME/KB3.2 (41%) and at the species level with Microflex/FilFungi V5 (21%).

### Comparison of instruments: VITEK MS, VITEK MS PRIME, and Microflex

As MSI-2 was the only database used with the MS, we looked at the MSI-2 rates of identified isolates in order to compare the three instruments (Table 2).

Regarding *Aspergillus* spp. and rare molds, there was no significant difference between the rates of identification with Microflex and bioMérieux’ instruments at the genus, section, or species levels (Table 2). However, for uncommon yeasts, Microflex performed significantly better than VITEK MS and VITEK MS PRIME at the genus and species level (*P* = 0.0002 and *P* = 0.0005, respectively).

Considering the identified isolates, there was no significant difference between VITEK MS and VITEKMS PRIME (Table 2). We could not obtain analyzable spectra (“Bad spectrum”) for 26 strains (15%, 11 uncommon yeasts, six *Aspergillus* spp., and nine rare molds) using the VITEK MS PRIME versus only five strains (3%, three uncommon yeasts, and two rare molds) with the VITEK MS (*P* = 0.005).

### Comparison of the four databases regarding the rate of correct identification

When the identification result reached the threshold, it either matched the expected one (correct identification) or not (misidentification).

For uncommon yeasts, at the genus level, the RCI ranged from 96% (VITEK MS PRIME/KB3.2 and KB3.3) to 100% (Microflex/FilFungi V5, VITEK MS PRIME/MSI-2, Microflex/MSI-2). At the species level, it ranged from 87% (VITEK MS PRIME/KB3.2 and KB3.3) to 100% (Microflex/FilFungi V5, VITEK MS PRIME/MSI-2) (Table 2).

For *Aspergillus* spp., at the genus level, the RCI ranged from 97% (Microflex/FilFungi V5) to 100% with (all remaining instrument/database combinations). At the section level, it ranged from 93% (Microflex/FilFungi V5) to 100% (all remaining instrument/database combinations) and at the species level from 50% (Microflex/FilFungi V5) to 77% (VITEK MS/MSI-2) (Table 2).

For rare molds, at the genus level, the RCI ranged from 97% (Microflex/FilFungi V5) to 100% (all other instrument/database combinations). At the species level, it ranged from 60% (Microflex/FilFungi V5) to 82% (VITEK MS PRIME/MSI-2) (Table 2).

When considering only the genera and species included in the KB databases, the increase in correctly identified strains was significant for uncommon yeasts (species), *Aspergillus* spp. (section and species), and rare molds (genus and species). For FilFungi V5, a significant increase was observed only for *Aspergillus* spp. (section and species) and rare molds species (Table 3). For MSI2, the rate of correctly identified species did not vary significantly, regardless of the instrument.

**TABLE 3:** Evolution of the rates of identified strains when restricting genus, Section (for *Aspergillus* spp. only) and species to those included in the databases KB3.2, KB3.3, and FilFungi V5^*c*^

	Type	Genus (%)	Genus restricted (%)	Section (%)	Section restricted (%)	Species (%)	Species restricted (%)
KB3.2^*a*^	Uncommon Yeasts	91	91	NA		91	94
	*Aspergillus* spp.	77	77	77	100	77	100
	Rare molds	50	84	NA		50	97
	All fungi	73	84	NA		73	97
KB3.3^*a*^	Yeasts	88	88	NA		88	94
	*Aspergillus* spp.	84	84	84	98	84	98
	Rare molds	63	89	NA		63	99
	All fungi	78	87	NA		78	97
FilFungi V5^*b*^	Yeasts	83	83	NA		61	67
	*Aspergillus* spp.	51	51	23	61	23	61
	Rare molds	47	56	NA		61	64
	All fungi	60	63	NA		35	64

^*a*^ Spectra acquired with VITEK MS.

^*b*^ Spectra acquired with Microflex.

^*c*^ NA, not applicable.

### Misidentifications

Out of 1,690 independent identifications performed on the three instruments and four databases, 219 misidentifications (i.e., misidentified isolates among those reaching the identification thresholds) were recorded. All instruments and databases combined, four misidentifications were recorded at the genus level and two at the *Aspergillus* spp. section level. Most of the misidentifications, 213 (213/219, 97%), occurred at the species level (Table S2). For uncommon yeasts, the highest misidentification rates at the species level were obtained with KB databases (13% for VITEK MS PRIME/KB3.2 and KB3.3).

For *Aspergillus* spp., the highest misidentification rates at the species level were obtained with KB databases (up to 43% with VITEK MS PRIME/KB3.3 and KB3.2). For rare molds, there was no significant difference between the databases, with the misidentification rates ranging from 17% (VITEK MS/MSI-2) to 29% (VITEK MS/KB3.2).

## DISCUSSION

Implementing MALDI-ToF MS in a routine work lab process would speed up the identification of yeasts and FF. This method is particularly interesting when mycologists are unable to provide species identification based on microscopic and morphological criteria and particularly appropriate to restrict the cost generated by DNA-based methods. At the beginning of MALDI-ToF use for FF, commercial databases were RUO and were outperformed by in-house databases (10). Nowadays, bioMérieux and Bruker are enriching the databases associated with their MALDI-ToF apparatus, some of them being certified for IVD, such as bioMérieux’s KB 3.2. The aim of this study was to compare those commercial databases to MSI-2, an open-access software for mass spectrometry identification (8), as well as challenging the novel VITEK MS PRIME device with uncommon yeasts, *Aspergillus* spp., and rare molds, all molecularly identified.

According to our panel, the most complete database is MSI-2, where 92% of the species tested were represented, followed by FilFungi V5 (59%), KB3.3 (58%), and KB3.2 (53%).

Identification results after 3 days of growth were significantly better than after a prolonged incubation of 8 days. Protein patterns vary along the fungal growth, leading to changing mass spectra (11). The influence of growth on MALDI-ToF MS identification seems to depend on the fungal genus (3). However, our results tend to prefer younger material. This is coherent with the fact that the databases are built with young fungal colonies, allowing for faster identification in the lab. Bruker recently proposed a direct deposit process for FF, which uses young mycelium, as their database is constructed with young colonies (12).

No significant difference could be observed between bioMérieux’ instruments in terms of rates of identification. The difference lies in the rates of spectra recognized as non-analyzable by the acquisition system, which are higher for VITEK MS PRIME than for VITEK MS. The VITEK MS PRIME seems to be more stringent, requiring increased spectral quality. If used in a daily routine, the VITEK MS PRIME would require careful attention on direct protein extraction for yeasts and on the quality and intensity of the laser. A default in those parameters translates itself into an increased number of “bad spectrum” outputs, which would need to be fixed by targeting a fine-tuning procedure of the laser. Using MSI-2 for yeasts, Microflex was more accurate than VITEK MS and VITEK MS PRIME. In our study, a complete extraction was performed before acquisition by Microflex, whereas only a direct deposit was realized on bioMérieux’s targets. Indeed, the MSI-2 database has been built with spectra acquired from fungal extractions (7). Our results are consistent with a previous multicenter study comparing three deposition protocols for yeast, using Bruker’s database: the direct deposition yielded lower performance compared to complete extraction (2). Therefore, complete protein extraction remains mandatory unless the manufacturer specifically recommends an alternative protocol.

On the other hand, no difference was observed with *Aspergillus* spp. and rare molds between Bruker and bioMérieux instruments. MSI-2 seemed capable of handling spectra from the three instruments with the same accuracy, which had not been shown before, as most of the literature data on MSI-2 was obtained using Microflex. This is of importance as MSI-2 is constructed with spectra from Bruker instruments; laboratories equipped with a VITEK MS or a VITEK MS PRIME are able to use MSI-2 as well.

When comparing the databases (KB3.2, KB3.3, Fungi V5, and MSI-2), the highest rates of identification at species level are obtained for uncommon yeasts with VITEK MS PRIME/KB3.2 and KB3.3 (92%). This is consistent with previous studies aiming at establishing MALDI-ToF performance for yeasts (13). We showed that this performance is conserved even when using uncommon species, consistent with a 2012 study which challenged an in-house database in differentiating *Cryptococcus gattii* and *Cryptococcus neoformans (*14).

When comparing the RCI for *Aspergillus* spp., at species level, MSI-2 outperformed bioMérieux and Bruker’s databases. With MSI-2, RCI reached up to 77% at species level, while KB3.3 achieved 60%. In 2019, a study used the first version of MSI (MSI-1) and achieved RCI of 100% at section level and 92.2% at species level for non-cryptic species and 66.1% for cryptic species (15). For *Aspergillus* spp., MSI-2 achieved 77% correct identification, concordant with this study, considering that our panel is composed mostly of cryptic species (15). In Barker et al*.*, performances were higher using MSI-2, reaching a 100% RCI at species level but using only non-cryptic species (3). Using IVD KB3.2, Zvezdánova et al. (16) achieved 85% RCI at species level, also using mostly non-cryptic species. Notably in our panel, numerous discrepancies occurred among the *Nidulantes* and the *Nigri* sections, which are known to contain species difficult to distinguish molecularly. Specifically, we considered *Aspergillus welwitschiae* and *Aspergillus niger* as two separate species, although this is discussed from a taxonomic point of view.

Sun et al*.* (17) in 2021 showed that IVD KBs are more efficient than Bruker’s database. In their study, VITEK MS identified 96% of their strains at species level, whereas MBT identified only 42%. Our results lead to the same conclusion, regardless of the fungal type, when comparing KB3.3 to FilFungiV5.

MSI-2 remains the best database to identify *Aspergillus* species and molds according to our results. This study used a panel composed mostly of rare molds and cryptic *Aspergillus* species. This explains why the RCIs, mostly at species level, are not perfect. Indeed, when only the species represented in the databases are considered, RCI increases (for bioMérieux’s commercial databases, RCIs are significantly higher). This shows that the comparing algorithm is able to analyze the spectra in order to give an identification with an acceptable confidence score when the species is referenced in the database. The main pitfall remains that all databases are incomplete by definition and that users will always encounter uncommon species that are not included in the databases. Indeed, common pathogens encountered in a daily routine are well represented in all commercial databases. The performances of the identification of these frequent pathogens are known to be good across all the commercial systems (17).

Considering misidentifications, among *Aspergillus* species, as only three occurred at section level, they consisted in discrepancies among the same section, between cryptic species. This has already been shown by Imbert et al. (15) in 2019, reporting an 87% misidentification among *Aspergillus* corresponding to cryptic species. Most of these misidentifications were not clinically significant in the way that the antifungal drug given to the patient would not have been changed with the correct identification. However, for the understanding of the infection, providing the right identification is of utmost importance, and this should be constant over time if infection is recurrent.

### Conclusion

Having optimized their instruments and their databases, nowadays, the commercial systems provide good performances in the identification of common fungal pathogens. On a daily routine, they might be sufficient, especially for frequent pathogens. As the comparing algorithms have also been optimized, currently the main pitfall is the incomplete databases. MSI-2 remains the best database for uncommon pathogens, as it is the most complete. Therefore, enhancing databases in collaboration with taxonomists will be manufacturers’ prime concern. MSI-2 can be used with both bioMérieux or Bruker instruments. As total extraction is time-consuming, technical improvements to speed up the workflow might be interesting, such as direct deposit. Using this method will require updates to acquisition systems and databases, as the resulting spectra are quite different from those obtained when performing a complete extraction.

## SUPPLEMENTAL MATERIAL

Table S1 jcm.01612-24-s0001.xlsx Aggregated identification rates per species analyzed in our study.

Table S2 jcm.01612-24-s0002.docx List of the misidentifications obtained in our study among the 1,690 independent identifications performed on all systems and databases.

ASM does not own the copyrights to Supplemental Material that may be linked to, or accessed through, an article. The authors have granted ASM a non-exclusive, world-wide license to publish the Supplemental Material files. Please contact the corresponding author directly for reuse.
