# Supplementary material for: Comparison of MALDI-TOF MS instruments and databases for the identification of uncommon yeasts, Aspergillus spp. and rare filamentous fungi
Source: J Clin Microbiol. 2025 May 15;63(6):e01612-24. doi: 10.1128/jcm.01612-24 (PMC12153315; doi:10.1128/jcm.01612-24)
Supplement: Table S2 — List of the misidentifications obtained in our study among the 1,690 independent identifications performed on all systems and databases. [file jcm.01612-24-s0002.docx]

**Supplemental Table 2:** List of the misidentifications obtained in our study among the 1,690 independent identifications performed on all systems and databases
